# Novel, non-invasive markers for detecting therapy induced neuroendocrine differentiation in castration-resistant prostate cancer patients

**DOI:** 10.1038/s41598-021-87441-2

**Published:** 2021-04-15

**Authors:** Divya Bhagirath, Michael Liston, Theresa Akoto, Byron Lui, Barbara A. Bensing, Ashok Sharma, Sharanjot Saini

**Affiliations:** 1 Department of Biochemistry and Molecular Biology, Augusta University, 1410 Laney Walker Boulevard, Augusta, GA 30912 USA; 2 Veterans Affairs Medical Center, San Francisco, USA; 3 Department of Cellular Biology and Anatomy, Augusta University, Augusta, USA; 4 Department of Population Health Sciences, Augusta University, Augusta, USA

**Keywords:** Cancer, Molecular biology, Biomarkers, Oncology, Urology

## Abstract

Neuroendocrine prostate cancer (NEPC), a highly aggressive variant of castration-resistant prostate cancer (CRPC), often emerges upon treatment with androgen pathway inhibitors, via neuroendocrine differentiation. Currently, NEPC diagnosis is challenging as available markers are not sufficiently specific. Our objective was to identify novel, extracellular vesicles (EV)-based biomarkers for diagnosing NEPC. Towards this, we performed small RNA next generation sequencing in serum EVs isolated from a cohort of CRPC patients with adenocarcinoma characteristics (CRPC-Adeno) vs CRPC-NE and identified significant dysregulation of 182 known and 4 novel miRNAs. We employed machine learning algorithms to develop an ‘EV-miRNA classifier’ that could robustly stratify ‘CRPC-NE’ from ‘CRPC-Adeno’. Examination of protein repertoire of exosomes from NEPC cellular models by mass spectrometry identified thrombospondin 1 (TSP1) as a specific biomarker. In view of our results, we propose that a miRNA panel and TSP1 can be used as novel, non-invasive tools to identify NEPC and guide treatment decisions. In conclusion, our study identifies for the first time, novel non-invasive exosomal/extracellular vesicle based biomarkers for detecting neuroendocrine differentiation in advanced castration resistant prostate cancer patients with important translational implications in clinical management of these patients that is currently extremely challenging.

## Introduction

Prostate cancer (PCa) is the second leading cause of cancer-related death among men in the United States^1^, with most of the mortality resulting from aggressive, metastatic disease. This malignancy is androgen-dependent acting primarily via binding to Androgen receptor (AR), leading to oncogenic signaling^2^. Therefore, ablation of AR signaling by androgen deprivation is the goal of first-line therapy^2^ that leads to tumor regression. However, in a significant fraction of patients, 2–3 years post-androgen deprivation, the disease progresses to castration-resistant prostate cancer (CRPC)^3^. Treatment options for CRPC has expanded in recent years with the introduction and approval of several new agents such as second generation of AR pathway inhibitors (API) enzalutamide (MDV3100/ENZ) and abiraterone (ABI)^2,4,5^. These agents have shown significant improvements in overall patient survival. However, resistance is near universal owing to heterogeneous molecular mechanisms such as AR bypass signaling or complete AR independence^6,7^. The latter leads to conversion of API-resistant prostate adenocarcinomas to AR independent variant of PCa^8^, referred to as neuroendocrine prostate cancer (NEPC), via a reversible trans-differentiation process known as neuroendocrine differentiation (NED). As a result of NED, PCa cells undergo lineage switching and exhibit characteristics of neuroendocrine (NE) cells, such as expression of neuronal markers including enolase 2 (ENO2), chromogranin A (CHGA) and synaptophysin (SYP)^9,10^. Owing to loss of AR signaling, these patients present with low serum prostate-specific antigen (PSA). Despite low serum PSA, the disease exhibits a more aggressive course, often clinically manifesting as high visceral metastatic burden including metastasis to liver, lung, central nervous system or bone. NEPC has poor prognosis with limited treatment options and a 5 year survival rate of < 20%^11^ and evolves via clonal evolution from adenocarcinomas in ~ 20% of CRPC cases^12^ via molecular events such as loss of tumor suppressors retinoblastoma (*RB1*) and tumor protein 53 (*TP53*). In addition, a series of key alterations at genetic and epigenetic levels^13^ occur including loss of phosphatase and tensin homolog (*PTEN*), frequent *TMPRSS2-ERG* gene rearrangements^14^, amplifications of Aurora kinase A (*AURKA*), *NMYC*, EZH2^12,15–17^ and neural transcription factors BRN2^18^ and BRN4^19^. NEPC can also arise de novo from NE cells of normal prostate gland though these cases are rare (1%)^6,20^. It is believed that de novo NEPC and treatment emergent NEPC evolve via distinct mechanisms, with the latter arising as a result of NED from adenocarcinomas in metastatic CRPC, primarily as a result of prolonged hormonal therapy, though it can arise in some cases after primary docetaxel therapy or even early on after API treatment^9,10,21^.

Currently, there is a lack of effective molecular biomarkers for predicting API therapy resistance and emergence of therapy-induced NEPC^9,10^ in CRPC patients. Histopathological assessment of biopsy tissues is currently used for NEPC diagnosis. Though neuronal markers including SYP, ENO2, CHGA and CD56 have been used to monitor API-induced NEPC via expression analyses in biopsy tissues or serum samples^10^, these markers lack specificity. Though elevated serum CHGA levels have been shown to correlate with shorter progression-free survival following abiraterone treatment^22^, a recent study showed that its levels are unable to discriminate between CRPC and NEPC^21^. NEPC often manifests in patients with multiple metastases that hinders clinicians from performing invasive biopsies. As a result, NEPC is often underdiagnosed. NEPC is refractory to AR targeted therapies and is treated with cisplatin/carboplatin combinations with either docetaxel or etoposide^23^. However, in absence of effective diagnosis, these cases fail to be treated with appropriate therapies leading to poor survival. Thus, it is highly imperative to identify novel, non-invasive molecular markers to diagnose the emergence of NEPC in CRPC patients.

Recently, exosomes/extracellular vesicles (EVs) have emerged as a source of alternate, non-invasive, disease biomarkers that are detectable in biological fluids such as serum, plasma and urine (30). Exosomes are small membranous EVs, 30–150 nm in size, (29) that are shed from living cells upon fusion of the multivesicular bodies with the plasma membrane^24,25^. It is now being increasingly recognized that the cells selectively package functional biomolecules in these vesicles and that these vesicles contain proteins, RNAs (including microRNAs) and lipids. EV cargo is often reflective of the physiological state of the originating host cell, varying under various pathological conditions, including cancer^26^. An increased number of exosomes are typically secreted by cancer cells, often correlating between cancer stage and progression^27^ as these vesicles mediate intercellular communication and execute important functions in tumor biology such as induction of proliferation, angiogenesis and metastatic development^28^. EVs/exosomes can be used as liquid biopsy for various cancers, including PCa (31–33). MicroRNAs (miRNAs), small non-coding RNAs that suppress gene expression post transcriptionally via sequence-specific interactions with the 3′- untranslated regions (UTRs) of cognate mRNA targets^29^, are stable biomarkers that are abundantly represented in exosomes^30,31^. It has been reported that these vesicles provide an enriched source of miRNAs for biomarker profiling by protecting against RNases as compared to intracellular miRNAs/ miRNAs present in cell-free blood^32^. The goal of the present study was to identify novel, non-invasive, EV-based microRNA/protein biomarkers for diagnosing treatment-induced NED in CRPC patients.

## Results

### Dysregulation of EV-miRNA content as CRPC cells undergo NED

**Figure 1:**
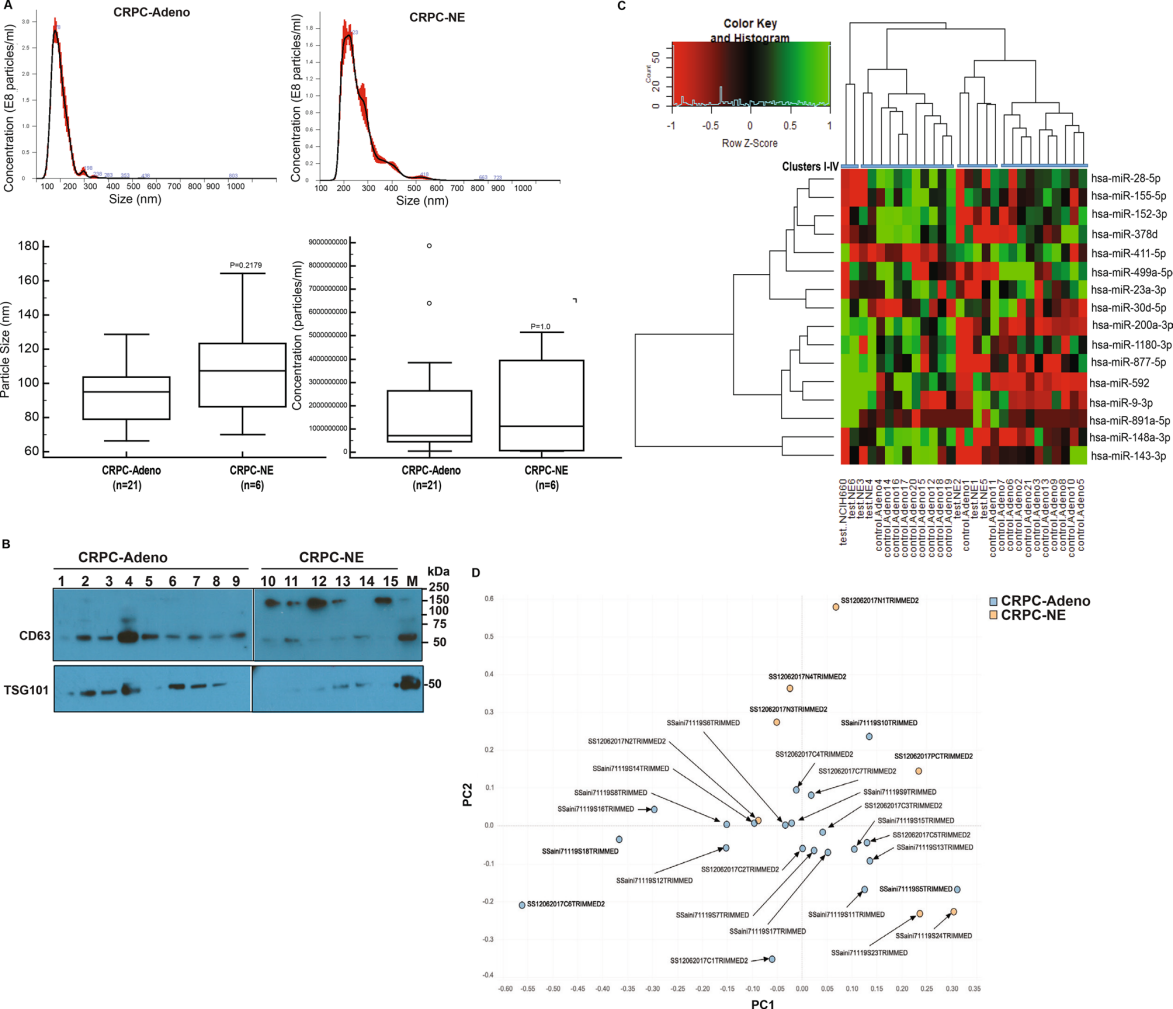
miRNA profiling of exosomes/EVs isolated from sera of CRPC-adenocarcinoma and CRPC-NE (treatment-induced) cases. (**A**) Representative NTA analyses for CRPC-Adeno (upper left panel) and CRPC-NE case (upper right panel). Particle size (left) and concentration (right) in CRPC-Adeno and CRPC-NE cases as determined by NTA analyses. (**B**) Western blot analyses for indicated exosomal markers in CRPC-Adeno and CRPC-NE cases. Samples 1–9 and 10–15 were run on two separate gels, with the partitioning indicated by solid black line. Samples derive from the same experiment and gels/blots were processed in parallel. Since CD63 and TSG101 fall in same size range, Western blots were initially probed with CD63 antibody. Following stripping, blots were re-probed with TSG101 antibody. (**C**) Heat map showing differentially expressed mature EV miRNAs in CRPC-adeno cases (n = 21) as compared to CRPC-NE (n = 6) cases. Heat map was generated by using R studio software, Version 1.1.463 (https://www.npackd.org/p/rstudio/1.1.463). Clusters (I-IV) are denoted by blue bars. (**D**) PCA plot showing EV-miRNA profiles in CRPC-Adeno and CRPC-NE cases.

We previously showed that progression of advanced CRPC with adenocarcinoma characteristics (CRPC-Adeno) to androgen-independent neuroendocrine states (CRPC-NE) is associated with a characteristic set of miRNA alterations in PCa tissues that drive change in cellular gene expression patterns towards NE states^33^. In view of this, we reasoned that exosomes/EVs released from PCa cells undergoing t-NEPC may be reflective of these alterations and can be a potential source of novel miRNA biomarkers. With a goal of examining miRNA alterations induced in exosomes/EVs upon induction of NED in CRPC, we purified exosomes/EVs from CRPC patients (Table S1) with adenocarcinoma characteristics (CRPC-Adeno, n = 21) and those with neuroendocrine differentiation (CRPC-NE, n = 6). Isolated EV preparations were comprehensively characterized by NTA (Fig. 1A) and immunoblot analyses for presence of exosomal markers CD63 and TSG101 (Fig. 1B). NTA analyses showed that the average particle size (Fig. 1A, lower left panel) and numbers (Fig. 1A, lower right panel) were higher in CRPC-NE cases as compared to CRPC-Adeno samples, though the differences failed to reach statistical significance. As a positive control, we included EVs extracted from NEPC cell line, NCI-H660 (30) that is derived from PCa lung metastasis (30). cDNA libraries were generated using an Illumina TruSeq small RNA library prep kit as per manufacturer’s instructions, equally pooled and sequenced on Illumina NextSeq 500 platform (Fig. 1C). Sequencing reads were adapter trimmed and analyzed. We obtained an average of ~ 8.5 million raw reads/library. ~ 95% reads were mapped to known RNA species and the human genome (hg38). Among the mapped miRNA reads, known mature miRNA reads were abundant (30–75%) followed by isomiRs corresponding to known precursors (30–40%), novel mature miRNA (~ 0.2–5%) and novel iso-miRs (0.2–2%). Raw sequencing data is deposited in the Sequence Read Archive at NCBI (accession no. SUB7247900). Significant dysregulation in miRNA expression patterns were observed between CRPC-Adeno vs CRPC-NE cases as represented in heat map in Fig. 1C. Unsupervised hierarchical clustering of miRNAs could cluster the analyzed clinical cases into four major clusters- I-IV with cluster I containing 100% NE samples, cluster II with 18% (2/11) NE cases and 82% adenocarcinomas, cluster III with 60% (3/5) NE cases and 40% adenocarcinomas while cluster IV lacked NE cases and comprised 100% of CRPC-Adenocarcinomas. These data suggest that while characteristic miRNA alterations are predominantly prevalent in CRPC-NE cases as compared to adenocarcinomas, treatment-induced NEPC cases exhibit enormous tumor heterogeneity that is reflected in derived EVs. Using a cutoff false discovery ratio (FDR) of 5% and adjusted P-value < 0.05, a total of 182 known and 4 novel miRNAs were found to be significantly differentially expressed between EVs from CRPC-Adeno vs CPRC-NE cases (Table S2). These differentially expressed miRNAs included 16 known mature miRNAs and 170 iso-miRs. The differentially expressed known mature miRNAs clustered largely into three distinct clusters of miRNA families (Fig. 1C). Among significantly dysregulated miRNA families, top downregulated miRNA families included miR-145, miR-148, miR-143 and miR-155 while top upregulated miRNA families were miR-1269, miR-153, miR-1468, miR-182 and miR-1301 (included in Table S2). Known mature miRNA alterations that were observed included upregulated miRs (miRs-891a-5p, -9-3p, -877-5p, -592-3p, -200a-3p) and downregulated miRNAs (miR-148a-3p, 143-3p, 378d, 499a-5p, 155-5p, 28-5p, 152-3p, 23a-3p, 1180-3p, 411-5p, 30d-5p) (Table S2). Alterations observed in iso-miRs are described in following section. Further, based on miRNA profiles (mature miRs + iso-miRs) of CRPC-Adeno vs CRPC-NE EVs, unsupervised analysis was performed using principal component analyses (PCA) (Fig. 1D) that showed separate clustering of the CRPC-NE tumors from CRPC-Adenocarcinomas primarily along PC2 for samples NE1-NE4 (denoted as sample name followed by N1-N4) and NCI-H660 (denoted as ‘PC’). NE5-NE6 (denoted as sample name S23 and S24) segregated from CRPC-Adeno EVs along PC1. These data suggest that specific miRNAs are dysregulated in induction of NE states and that miRNA expression patterns in EVs derived from these tumor states can largely distinguish between CRPC-Adeno and CRPC-NE tumors. This pattern of miRNA alterations observed in EVs were very specific as a similar analysis based on differential piwi-related RNAs (piRNAs) showed a non-distinct clustering of CRPC-NE with CRPC-Adeno tumors (Fig. S1).

### Predominant dysregulation of miRNA isoforms in EVs from NEPC

**Figure 2:**
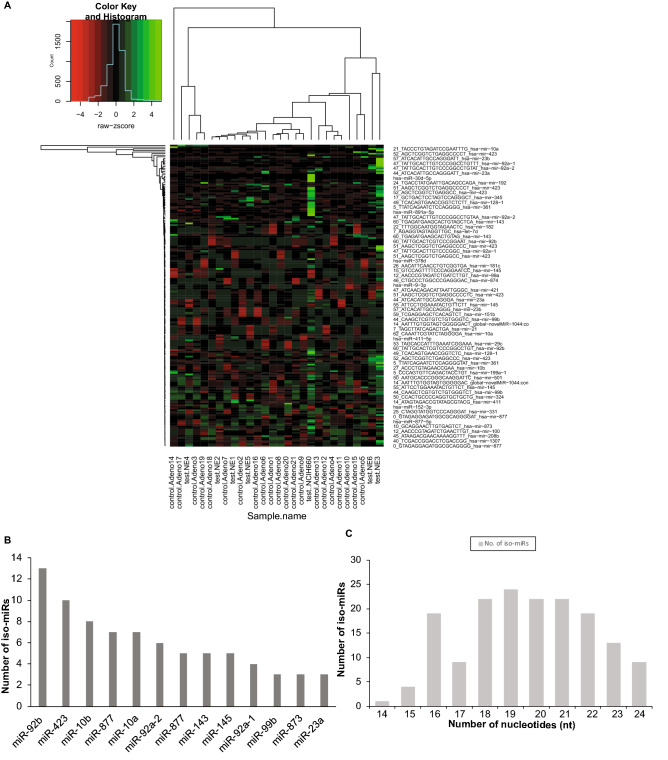
Predominant dysregulation of miRNA isoforms in EVs from NEPC. (**A**) Heat map showing differentially expressed iso-miRs in EVs from CRPC-adeno cases (n = 21) as compared to CRPC-NE (n = 6 clinical tissues + NCI-H660 cell line). Heat map was generated by using R studio software, Version 1.1.463 (https://www.npackd.org/p/rstudio/1.1.463). (**B**) miRNA loci producing ≥ 3 differentially expressed iso-miRs in sequenced samples plotted as a function of number of observed iso-miRs. (**C**) Range of length of differentially expressed iso-miRs (14–24 nucleotides) and their abundance across sequenced EV miRNAs extracted from CRPC-Adeno and CRPC-NE samples.

Notably, a significant representation of miRNA isoforms (iso-miRs) was observed in EVs, with a significant alteration in expression of a set of 170 iso-miRs (Fig. 2A and Table S2). Top significantly upregulated iso-miRs included those of miR-92b, -423, -10b, -10a, -877, -92a-1, -92-a2, 99b and downregulated miRs had a significant representation of isoforms of miR-143 and miR-145. Figure 2B represents the miRNA loci producing ≥ 3 differentially expressed iso-miRs with the predominance of iso-miRs from miR-92b, miR-423, miR-10a, miR-10b and miR-877. Iso-miRs were found to range in length from 14 to 24 nucleotides (nt) (Fig. 2C), with the majority of iso-miRs at 18-21nt. Interestingly, all the isoforms represented in EVs differ from native mature miRNAs in their 3′ regions with the seed sequence same as that of corresponding native miRNA.

### An EV-microRNA classifier of neuroendocrine differentiation in CRPC

**Figure 3:**
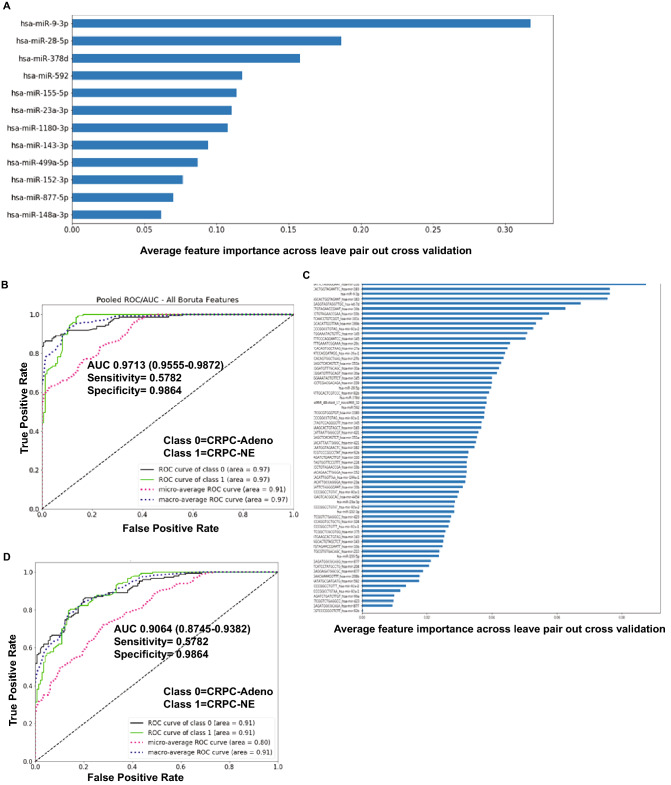
An EV-microRNA classifier of neuroendocrine differentiation in castration resistant prostate cancer. (**A**) Application of machine learning methods (random forest machine learning technique with leave-pair-out cross validation) to the NGS dataset of analyzed NE tissues + NCI-H660 cell line (CRPC-NE, n = 7) vs those with adenocarcinoma features (CRPC-Adeno, n = 21) yielded a ‘12 miRNA classifier’. miRNAs are listed in the order of feature importance as determined by these methods. (**B**) ROC curve analyses showing the ability of ‘EV-miRNA classifier’ to distinguish between class 0 (CRPC-Adeno) and class 1 (CRPC-NE). (**C**) An EV- miRNA classifier including isoforms of miRNAs as determined by random forest machine learning technique with leave-pair-out cross validation as applied to the NGS dataset of analyzed EVs from CRPC-NE (n = 6) + NCI-H660 cell line vs those from CRPC-Adeno patients (n = 21) including miRNA isoforms. miRNAs are listed in the order of feature importance. (**D**) ROC curve analyses showing the ability of ‘EV-miRNA classifier including iso-miRs’ to distinguish between class 0 (CRPC-Adeno) and class 1 (CRPC-NE).

Considering significant miRNA dysregulation patterns observed in CRPC-Adeno vs CRPC-NEs, we next explored if the observed differentially expressed EV-miRNAs can be used as a ‘molecular tool’ to differentiate between CRPC-NE and CRPC-Adenocarcinomas. To examine this, we employed random forest machine learning technique with leave-pair-out cross validation (LPOCV) to the NGS dataset of analyzed EVs from NE samples + NCI-H660 cell line (CRPC-NE) vs those with adenocarcinoma features. For this technique, miRNAs were filtered based on adjusted P-values and expression status to exclude those with P > 0.05 and low expressors. Also, miRNA isoforms (iso-miRs) were excluded. Interestingly, a set of 12 miRNAs formed an ‘EV miRNA classifier’ (Fig. 3A) that could distinguish CRPC-NE samples from CRPC-Adeno with an area under the ROC curve (AUC) of 0.9713 (Fig. 3B). Figure 3A depicts the miRNA classifier with the miRNAs listed in the order of feature importance as determined by LPOCV with top five features of the classifier including miRs-9-3p, -28-5p, -378d, -592 and miR-155-5p. Further, in view of observed significant dysregulation of iso-miRs in PCa EVs, we asked if incorporating iso-miRs in the classifier would be advantageous. Towards this, we applied a second machine learning method with iso-miRs included (Fig. 3C) that identified a ‘67 miRNA EV classifier’ that could distinguish CRPC-NE tissues from CRPC-Adeno with an AUC of 0.9064 (CI 0.8745–0.9382) (Fig. 3D), suggesting that while iso-miRs are significantly represented in PCa EVs, incorporating iso-miRs in the classifier does not improve its performance. Top features of the classifier included two miR-10b-3p isoforms, two of miRs-183 isoforms, one isoform of let-7d apart from typical miRNA miR-9-3p (Fig. 3C).

### Correlation of EV miRNA alterations with miRNA alterations in corresponding PCa tissues undergoing NED

**Figure 4:**
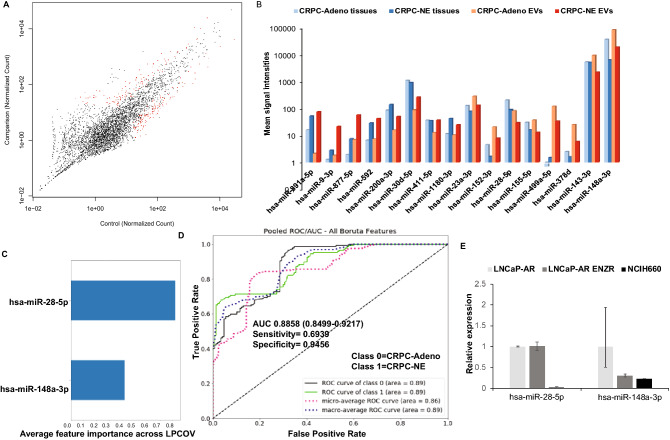
EV miRNA alterations in prostate cancer tissues undergoing NED. (**A**) Scatter plot showing EV-miRNA alterations in CRCP-Adeno vs CRPC-NE tissues profiled by small RNA NGS. (**B**) Plot comparing miRNA signal intensities of tissue and corresponding serum EV samples in CRPC-Adenocracinoma vs CRPC-NE cases. (**C**) Machine learning method as applied to EV-miRNA alterations that were also observed in corresponding prostate cancer tissues. (**D**) ROC curve analyses showing the ability of two-EV-miRNA classifier to distinguish between class 0 (CRPC-Adeno) and class 1 (CRPC-NE). (**E**) Validation of two-EV-miRNA classifier in NEPC cellular model NCI-H660 compared to parental LNCaP-AR and LNCaP-AR ENZ resistant cells by real time PCR based expression profiling.

We recently demonstrated that induction of PCa NED is accompanied by key alterations in microRNA repertoire of PCa tissues and that a ‘miRNA signature’ derived from these tissues have potential diagnostic value in assessing NED in CRPC patients^33^. We next asked as to how the dysregulated EV-miRNAs correlate with the miRNA alterations in corresponding tissues as serum EVs could be derived from diverse cellular sources. To examine this, we performed small RNA NGS in microdissected CRPC-Adeno (n = 21) vs CRPC-NE tissues (n = 6) from the same set of patients (Fig. 4A) that were employed for EV-miRNA profiling (Fig. 1) and compared the corresponding tissue and serum EV expression patterns of dysregulated miRNAs (Fig. 4B and Table S3). In addition, we also sequenced previously characterized patient-derived xenograft models representing CRPC-NE states—LuCaP 49, 145.1 and 145.2^33,35^ vs CRPC-Adeno PDXs LuCaP 70, 78, 81 and 92. Out of the observed EV-miRNA alterations, miR-891a-5p, -9-3p, -877-5p, -592, -200a-3p, 1180-3p were found to be similarly upregulated while miR-152-3p, -28-5p, -378d, -23a-3p were found to be decreased in CRPC-NE tissues similar to alterations in exosomes/EVs. miR-155-5p, -499a-5p and -148a-3p were found to exhibit opposite patterns in tissues and corresponding EVs (Fig. 4B). Amongst these alterations, only three miRNAs were significantly altered in both PCa tissues and EVs: miR-1180-3p, miR-148a-3p and miR-28-5p. miR-148-3p was found to be significantly decreased in EVs from CRPC-NE cases while the corresponding tissues showed an increased expression of this miRNA in CRPC-NE tissues as compared to CRPC-Adenocarcinomas. We next asked if we can derive a miRNA classifier based on these significant miRNA alterations that are also observed in corresponding tissues. Towards this, we performed a second machine learning method, restricting to EV-miRNA alterations that were also observed in concomitant tissues (Fig. 4C) and using this method, we found a two-miRNA classifier, composed by miR-28-5p and miR-148a-3p that could distinguish between CRPC-Adeno and CRPC-NE states with an AUC of 0.8858 (Fig. 4D). This suggests that though a combination of miRNAs have a better predictive potential than a set of two miRNAs. However, the two-miRNA classifier may be more meaningful as it is truly reflective of alterations associated with NEPC.

### Validation of two-miRNA classifier in NEPC cellular models

We further asked if the deduced EV-miRNA classifier from sera is a valid NEPC classifier. To examine this, we extracted EVs from NEPC cellular model NCI-H660 alongwith LNCaP-AR and LNCaP-AR-EnzR cell lines. Following extensive EV characterization, miRNAs were extracted followed by profiling by real-time PCR (Fig. 4E). Our profiling showed that miR-28-5p and miR-148a-3p are downregulated in EVs from NCI-H660 cells as compared to EVs from LNCaP-AR cells, validating the association of low expression of these miRNAs with NEPC. LNCaP-AR-Enz resistant cell line showed downregulation of miR-148a-3p while miR-28-5p levels were not significantly altered.

### EV-miRNA profiling of de novo NEPC shows distinct miRNA alterations from treatment-induced NEPC

**Figure 5:**
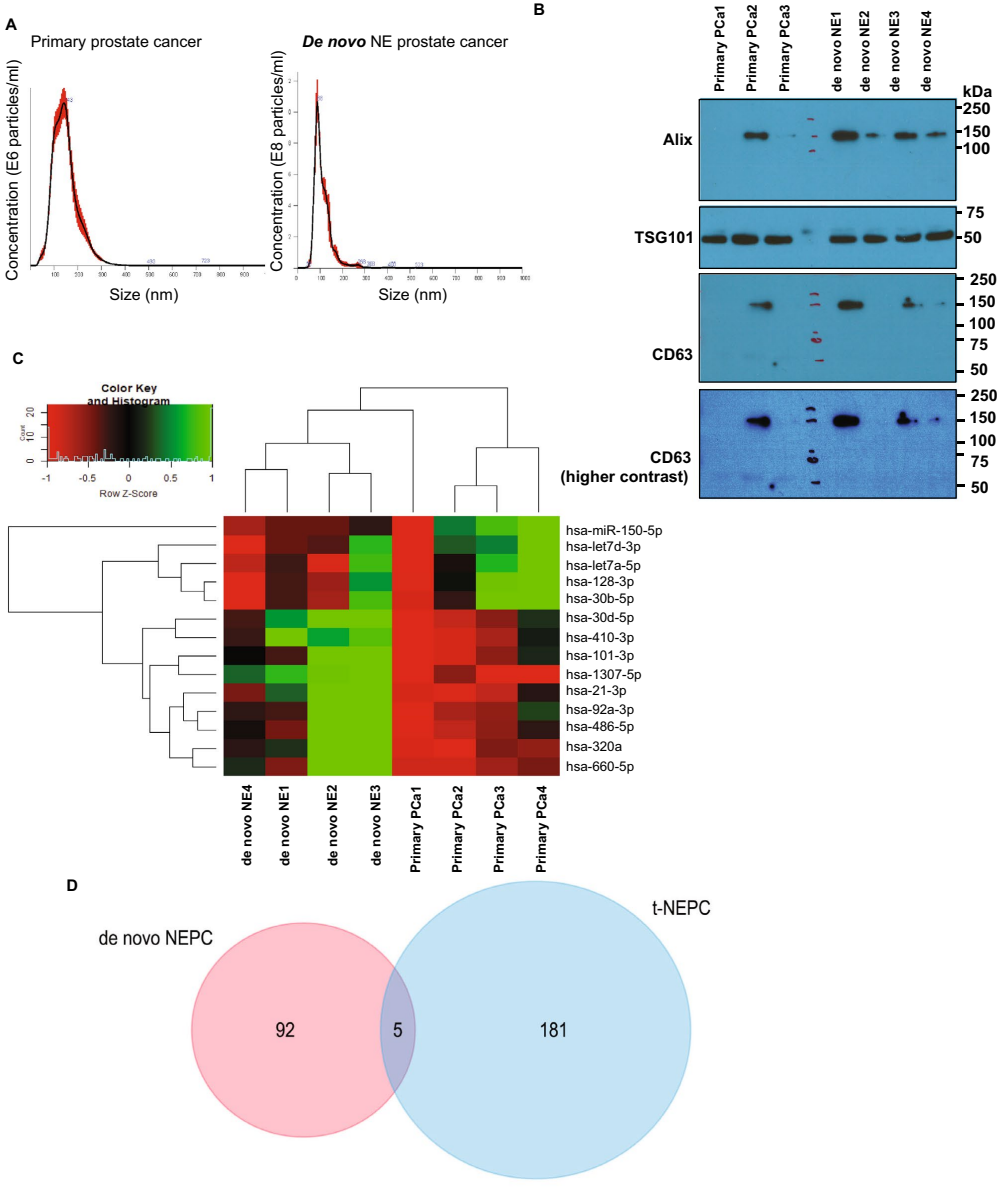
EV-miRNA profiling of de novo NEPC shows distinct miRNA alterations from treatment-induced NEPC. (**A**) Representative NTA analyses of EVs isolated from primary adenocarcinomas (left) and de novo NEPC (right). (**B**) Western blot analyses for exosomal markers in EVs isolated from primary adenocarcinomas and de novo NEPC. Following transfer of gel, blots were cut according to molecular weights. Upper parts of blots were probed with Alix antibody and lower parts were probed with TSG101 antibody. Since CD63 and TSG101 fall in same size range, following stripping, blots were re-probed with CD63 antibody. (**C**) Heat map showing differentially expressed miRNAs in EVs from de novo NEPC patients and those with primary adenocarcinomas. Heat map was generated by using R studio software, Version 1.1.463 (https://www.npackd.org/p/rstudio/1.1.463). (**D**) Venn diagram showing miRNA altered significantly in de novo NEPC vs treatment induced NEPC.

We were interested in examining the correlation between miRNA alterations represented in PCa EVs upon treatment-induced NEPC and de novo NEPC tumors. Towards this, we extracted EVs from de novo NEPC patients (Fig. 5) (n = 4) and primary prostate adenocarcinomas (n = 4). Following characterization of EVs by NTA and Western blotting for exosomal markers (Fig. 5B), EV-miRNAs were extracted followed by small RNA NGS on Illumina NextSeq 500 platform (Fig. 5C and Table S4). This profiling revealed a significant dysregulation of a set of 97 miRNAs, including 83 iso-miRs and 16 miRNAs. Significant miRNA alterations included upregulation of miR-660-5p, miR-410-3p and downregulation of miR-150-5p and miR-128-3p, among others. A comparison of differentially expressed miRNAs in EVs from therapy-induced NE samples and de novo NE cases showed that there is little overlap in their EV-miRNA repertoire (Fig. 5D). Only miR-30d-5p and three iso-miRs (one each of miR-23a, miR-23b, miR-345 and 92a-2) were found to be common miRNAs dysregulated between the two datasets suggesting that EV alterations in de novo NEPC and therapy-induced NEPC are distinct.

### Mass spectrometric analyses of protein content of EVs from NEPC cellular models

**Figure 6:**
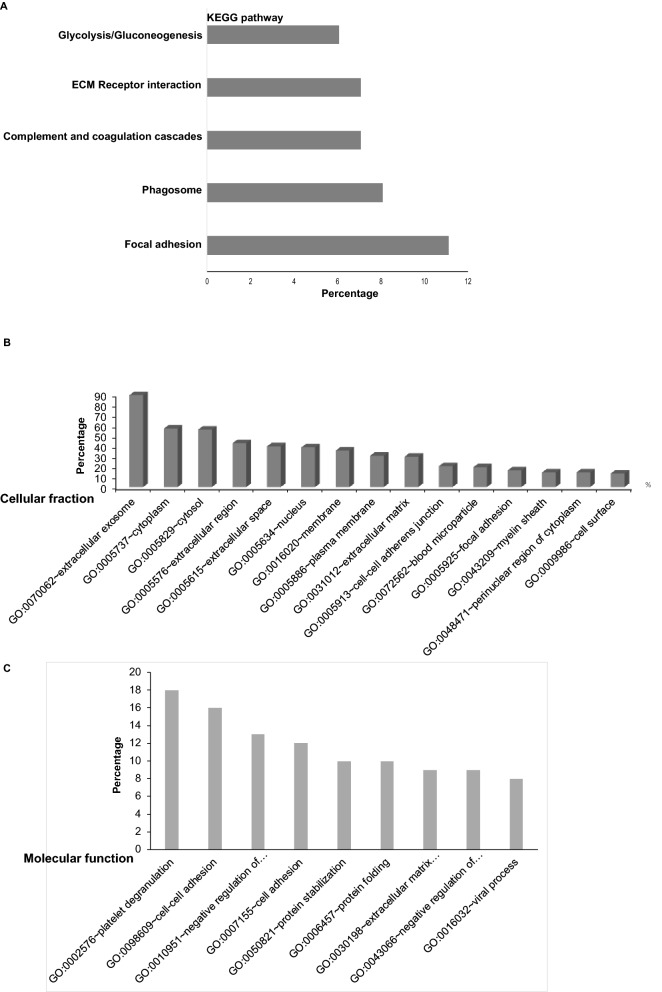
Mass spectrometric analyses of protein content of EVs from NEPC cellular models. Following extensive characterization of EVs, proteins were isolated from LNCaP-AR, LNCaP-AR-EnzR and NCI-H660 cells followed by mass spectrometric analyses by Shot gun approach and analyses by (DAVID) v 6.8 software^63^ to discover the association of identified proteins with biological processes, cellular components and KEGG pathways^36,37^. (**A**) KEGG pathways^36,37^ impacted by proteins isolated from EVs of NCI-H660 cells as compared to EVs from LNCaP-AR cells. (**B**) Cellular fraction of EV proteins from NCI-H660 cells as compared to EVs from LNCaP-AR cells. (**C**) Molecular function of EV proteins from NCI-H660 cells as compared to EVs from LNCaP-AR cells.

**Figure 7:**
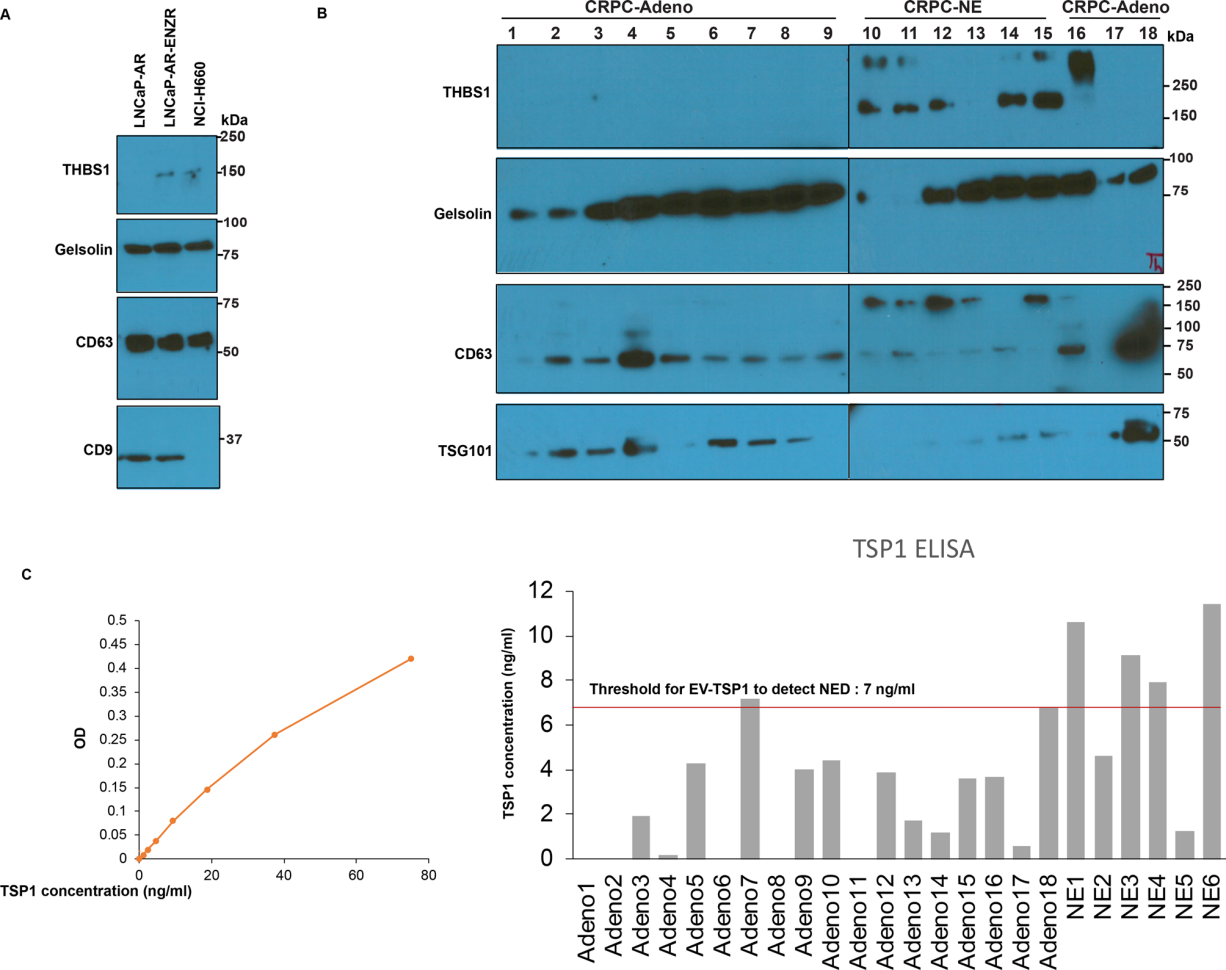
Thrombospondin 1 as a novel, highly specific EV protein biomarker for neuroendocrine prostate cancer. (**A**) EV-associated TSP1, Gelsolin and exosomal markers CD63 and CD9 expression as analyzed by Western blotting in EVs from LNCaP-AR, LNCaP-AR-EnzR and NCI-H660 cells. (**B**) EV-associated TSP1, Gelsolin and exosomal markers CD63 and TSG101 expression as analyzed by Western blotting in EVs from CRPC-Adeno and CRPC-NE tissues. Clinical samples were run in two separate gels (lanes 1-9, gel no. 1 and lanes 10-18, gel no. 2). The boundaries of gels are delineated by black lines. Samples derive from the same experiment and gels/blots were processed in parallel. Following transfer of gel, blots were cut according to molecular weights. Upper parts of blots were probed with THBS1 antibody and lower parts were probed with Gelsolin and CD63 antibody. Since CD63 and TSG101 fall in same size range, following stripping, CD63 blots were re-probed with TSG101 antibody. (**C**) TSP1 ELISA to validate TSP1 as a novel NEPC biomarker. Left panel shows the standard curve derived from known concentrations of standard TSP1. Right panel shows computed TSP1 values from TSP1 ELISA assay using EVs from CRPC-Adeno and CRPC-NE clinical samples.

Further, we examined the protein content of EVs from LNCaP-AR, LNCaP-AR-Enz resistant and NCI-H660 cell lines. Following extensive characterization of EVs (Fig. S2 and Fig. 7A), proteins were isolated followed by mass spectrometric analyses by shot gun approach (Fig. 6). Our analyses identified several differentially expressed proteins in LNCaP-AR-Enz resistant and NCI-H660 EVs as compared to LNCaP-AR EVs (Table S5). Top downregulated proteins include Fibronectin 1, Heat shock protein 90α, actin cytoplasmic 2 and thrombospondin 1. Gelsolin was found to be increased in LNCaP-AR EnzR cells though it shows a decrease in NCI-H660 EVs. Top upregulated proteins include Breast cancer type 2 susceptibility protein (BRCA2), Septin 2, Activated RNA polymerase II transcriptional coactivator p15 and 60S ribosomal protein L10a. We further performed in silico analyses of cellular processes impacted by identified altered EV proteins by KEGG (Kyoto Encyclopedia of Genes and Genomes)^36,37^. Our analyses showed that focal adhesion, phagosome, ECM-receptor interactions, complement and coagulation cascades and glycolysis/gluconeogenesis are potentially impacted by altered protein in NCI-H660 EVs (Fig. 6A and Table S6). Further, 35% of EV proteins found in NEPC exosomes were predicted to be membranous, 56% cytoplasmic, 55% cytosolic, 38% nuclear, 20% were found to associated with cell cell adherens junction, 16% with focal adhesion and 13% were cell surface proteins (Fig. 6B and Table S6). In silico analyses of impacted biological processes showed that proteins involved in cell–cell adhesion, protein stabilization, protein folding, extracellular matrix organization and negative regulation of apoptotic process were highly represented (Fig. 6C and Tables S5 and S6).

### Thrombospondin 1 as a novel, highly specific EV protein biomarker for neuroendocrine PCa

Further, we sought to validate upregulated protein markers for NEPC by Western blotting (Fig. 7). Since thrombospondin1 and gelsolin were identified proteins by mass spectrometry, we performed Western blotting for these proteins alongwith exosomal markers in isolated EVs from LNCaP-AR, LNCaP-AR-EnzR and NCI-H660 cells (Fig. 7A). While Gelsolin was not altered in EVs from ENZ-R or NCI-H660 cells, thrombospondin 1 was found to be specifically upregulated in EVs from ENZ-R and NCI-H660 cells as compared to EVs from LNCaP-AR cells, validating THBS1 as a NEPC marker. To further validate this finding, we examined EVs from clinical CRPC-Adeno vs CRPC-NE cases (Fig. 7B) and observed a specific increase in EV-associated THBS1 in NE cases by Western blotting. In view of our results, we sought to examine if THBS1 ELISA can qualify as an assay for diagnosing NEPC. We tested THBS1 levels by ELISA in a cohort of CRPC-Adeno and CRPC-NE cases (Fig. 7C). Left panel shows the standard curve derived from known concentrations of standard THBS1. Right panel shows computed THBS1 values from THBS1 ELISA assay using EVs from CRPC-Adeno (n = 18) and CRPC-NE (n = 6) clinical samples. Importantly, a THBS1 threshold of 7 ng/ml serum could correctly categorize 17/18 (94%) tested samples as adenocarcinomas and 4/6 CRPC-NE (67%) samples as those with therapy-induced NED. These results consolidate the potential diagnostic value of EV-associated THBS1 for diagnosis of NEPC.

## Discussion

It is highly imperative to identify novel biomarkers to diagnose the emergence of t-NEPC in CRPC patients, that is currently challenging owing to the non-specificity of currently employed neuronal markers such as SYP, NSE, CHGA and CD56^10,21^. Our group recently reported that induction of PCa NED states is accompanied by key alterations in several miRNA families that drive neuronal gene expression patterns and that based on miRNA expression, CRPC-adenocarcinomas and CRPC-NE tumors could be distinctly stratified^33^. In this study, we examined the potential of EVs as a source of novel, non-invasive NEPC biomarkers. Our study shows that as prostate adenocarcinomas transition to NE states, EVs released from these cells undergo alterations in expression of specific miRNAs and proteins. Profiling of miRNA content of these EVs showed a significant dysregulation of key miRNAs and miRNA isoforms. Comparison of miRNA alterations in corresponding PCa tissues and EVs showed that several of these miRNA alterations were reflective of corresponding alterations in PCa tissues such as miR-891a-5p, -9-3p, -877-5p, -592, -200a-3p, 1180-3p were found to be upregulated while miR-152-3p, -28-5p, -378d, -23a-3p were found to be decreased in CRPC-NE tissues similar to alterations in exosomes/EVs. On the other hand, miR-155-5p, -499a-5p and -148a-3p were found to exhibit opposite patterns in tissues and corresponding EVs. EV profiles have been previously shown to be either reflective of alterations in host cells or opposite to that of host cells’ expression profiles^39^. It has been reported that cells utilize exosomes/EVs as a means of disposing off tumor suppressive miRNAs/components^40,41^ such as miR-23b in bladder cancer^41^, let-7 in metastatic gastric cancer cells^40^ and miR-1246 in prostate cancer^42^. Alternatively, exosomes carry information for intercellular communication as they transfer their miRNA/mRNA/protein cargo to adjacent/distant target cells^43,44^, allowing for rapid alterations in gene expression and control of critical processes such as growth, differentiation and cell survival, angiogenesis, and immunomodulation in target cells. We propose that observed EV-miRNAs drive intercellular communication in PCa promoting epigenetic reprogramming of prostate tumors towards NE states.

Importantly, we applied machine learning algorithms to our NGS dataset to identify a novel ‘EV-miRNA classifier’ that can be used to diagnose NED in CRPC patients non-invasively with important translational implications. Further, though the 2 miRNA classifier showed a lower AUC than ‘12 miRNA classifier’, the two miRNA classifier represents the true alterations in NEPC and may be more useful. Future studies with larger clinical cohorts are warranted to validate these findings. We believe that this ‘EV-miRNA classifier’ can provide significant advancement over current methods of assessing NED and can be used for predicting responsiveness to API inhibitors in the clinic. EV-miRNA biomarkers for NEPC have not been studied yet though exosomal miRNA biomarkers for mCRPC patients were examined by Huang et al*.* and miR-375 and miR-1290 were reported to be upregulated^38^.

Exosomal miRNAs and other contents are selectively sorted into exosomes via mechanisms that are yet to be fully understood^45^. Analysis of the miRNAs sequences presented in exosomes identified common seed sequences, termed EXO-motifs, that facilitates binding to RNA-binding proteins, such as hnRNPA2B1 and SYNCRIP^46,47^. In addition, cellular availability of miRNAs is a factor which determines the abundance of exomiRs. Alteration of cellular expression of mRNA targets for miRNAs have been reported to alter sorting of miRNAs into exosomes^48^. In our study, we observed that miR-148a exhibits decreased expression in exosomes/EVs while showing increase in corresponding tissues. Its decreased release in EVs, combined with decreased levels of miR-28 was found to be a predictor of PCa NED. miR-148a has been previously reported to be an androgen-regulated miRNA that promotes proliferation of PCa cells^49^ and is involved in attenuating paclitaxel resistance of hormone-refractory, drug-resistant PC3 cells by regulating mitogen and stress activated protein kinase (MSK1) expression^50^. In view of our results, we suggest that miR-148a expression is preferably retained in PCa cells where it may drive NE-specific alterations, contributing to its decreased release in EVs.

Importantly, we saw an overrepresentation of miRNA isoforms in EVs. IsomiRs differ from canonical sequences at the 5′end, 3′end or within the seed sequence and have been reported to target largely distinct set of mRNAs^51^ and can be highly specific and sensitive cancer biomarkers^52^. Addition or removal of nt at the 5′-end and changes within the seed sequence give rise to new seed sequences and therefore new mRNA targets, while changes at the 3′-end can affect stability^53^. The observed iso-miRs were mostly additions at 3′ end and not 5′ end with similar seed sequences suggesting that iso-miRs represented in exosomes mostly differ from native miRNAs in terms of stability. It has been reported previously that RNA with short half-lives are enriched in EVs, suggesting that their presence in EVs may reflect their reduced stability and increased turnover. Alternatively, isoforms represented in EVs may be involved in regulating the expression of a distinct set of mRNA/protein targets^51^, thereby impacting intercellular communication promoting tumor aggressiveness and induction of NED states. Future studies are needed to address the precise role/significance of iso-miRs observed in EVs. Interestingly, there was a prominent representation of miR-92b isoforms. hsa-miR-92b has been shown to be overexpressed, specifically in brain primary tumors, as compared to primary tumors from other tissues and brain metastases^54^. Further, it is not clear if therapy-induced NED is the same disease as de novo small cell PCa that emerges from rare neuroendocrine prostate cell populations^55^. Our data suggests that consistent with the notion that de novo NE tumors represent a state distinct from t-NEPC, miRNA alterations in EVs from de novo PCa were very different, with an overlap of only miR-30d and a set of three iso-miRs.

Importantly, our study suggests that EV-associated THBS1 is increasingly released in PCa exosomes upon NED induction and is a potential novel NEPC biomarker. Thrombospondin1 is an anti-angiogenic factor that was recently reported to be repressed in NEPC^34^. This study showed that NED and angiogenesis are both regulated by CREB (cAMP response element-binding protein) that enhances EZH2 activity that in turn, repressed THBS1 expression^34^. In breast cancer, it has been demonstrated that exosomal THBS1 facilitates the transendothelial migration of breast cancer cells via disrupting the intercellular integrity of endothelial cells^56^. We propose that EV associated THBS1 may drive tumor aggressiveness and NED in advanced PCa. While exosomal/EV THBS1 was found to be a highly specific marker for NEPC, it would be interesting to examine the combined potential of ‘miRNA classifier’ with ‘THBS1′ protein expression as a novel method for assessing NED in CRPC patients employing a large clinical cohort.

In conclusion, we identify for the first time, that induction of therapy-induced NED in advanced PCa is associated with significant alterations in miRNA and protein cargo in EVs. These alterations can be exploited for non-invasive monitoring of therapy-induced NED in CRPC patients. Importantly, we define for the first time, a novel, ‘EV-based miRNA classifier’ and THBS1 as a novel NEPC marker with potential translational implications. A limitation of our study was the small number of analyzed CRPC-NE samples. Currently, it is challenging to acquire ‘bona-fide’ CRPC-NE samples considering the rarity of these cases and also challenges in properly characterizing treatment-induced NEPC and differentiating those from adenocarcinomas based on expression of existing neuronal markers such as chromogranin A, synaptophysin and enolase 2. To circumvent this issue, we validated the two-miRNA classifier in NEPC cellular model NCI-H660. Similarly, thrombospondin 1 was validated as an EV-protein marker in NCI-H660 cells (Fig. 7A) in addition to clinical samples (Fig. 7B,C). Future studies validating the EV-miRNA classifier and THBS1 as a NEPC marker are warranted in larger cohorts. We believe that upon further validation, these markers can be employed in the clinic for diagnosing NED in CRPC patients and predicting response to APIs that is currently challenging.

## Methods

### Clinical samples

The study was conducted in accordance with ethical guidelines of US Common Rule and was approved by the UCSF and Augusta University committees on human research. Written informed consent was obtained from all patients. Serum samples (0.5–1 ml) and corresponding FFPE tissues were procured from PCa Biorepository Network (PCBN)/Co-operative Human Tissue Network (CHTN). CRPC-adeno (n = 21) included metastatic CRPC patients with no evidence of NED while CRPC-NE (n = 6) included metastatic AR- patients with therapy-induced NED (Table S1). Sera from de novo NEPC patients were obtained from Roswell Park Cancer Institute. Biospecimens were obtained within 8 h of death from patients who died of metastatic CRPC/NEPC. Serum samples were stored at − 80 °C till processed. Follow up clinical information (including prior therapies) was obtained for all the clinical samples.

### Cell lines and cell culture

NCI-H660 (CRL-5813)^57^ cell line was obtained from the American Type Culture Collection (ATCC) and cultured under recommended conditions in HITEs media supplemented with 5% FBS, and 1% penicillin/streptomycin. LNCaP-AR and LNCaP-AR-enz resistant cell lines were a kind gift from Dr. Felix Feng at UCSF and were maintained in RPMI 1640 media each supplemented with 10% fetal bovine serum (FBS) (Atlanta biologicals) and 1% penicillin/streptomycin. Enz resistant cells were cultured in presence of 50 µM enzalutamide (Selleck Chemicals). All cell lines were maintained in an incubator with a humidified atmosphere of 95% air and 5% CO_2_ at 37 °C.

### Isolation of exosomes

Serum-derived exosomes (EVs) were isolated from 250μL of serum using the Total exosome isolation reagent (Life Technologies, catalog number 4478360) as per manufacturer’s instructions and as described previously^19,42^. Briefly, serum samples were initially spun at 2000×*g* for 30 min to get rid of cells and debris. Next, 0.2 volume of exosome isolation reagent was added to each sample and samples were incubated at 2–8 °C for 30 min. The precipitated exosomes were recovered by centrifugation at 10,000×*g* for 10 min at room temperature.

For isolation of EVs from cell culture media, cells were grown in recommended media supplemented with 1% penicillin–streptomycin and 10% of exosome-depleted FBS (Gibco, catalog number A27208-01) at 37 °C, 5% CO_2_, 95% air for 48 h before collecting the conditioned medium. Cells were cultured in 10 cm dishes in 7 ml of media. At the time of collection of conditioned media, cells were ~ 50–60% confluent. All the following steps for EV/exosome isolation were carried out at 4 °C. Conditioned medium was centrifuged at 2000×*g* for 30 mins (Fisher Scientific accuSpin 1R, fixed angle rotor) and the supernatant was transferred to 10 K ultracel filter (Amicon ultra-15, catalog number UFC901024) to concentrate the supernatant. Following this step, 0.5 volume of total exosome isolation reagent (catalog number 4478359) was added to concentrated supernatant. After an overnight incubation at 2–8 °C, the supernatant was centrifuged at 10,000×*g* for 1 h (Fisher Scientific accuSpin micro 17R, fixed angle rotor). Pellets were re-suspended in 70–100µL of PBS and isolated EVs/exosomes were stored at -20 °C till further processing.

### EV quantitation and size determination

To confirm the integrity of EV preparations, particle sizes and concentrations were evaluated using nanoparticle tracking analysis (NTA) on a NanoSight LM10 instrument (Malvern Instruments) as per manufacturer’s instructions as described in^19,42^.

### RNA extraction from EVs, cultured cells and FFPE tissues

EV/exosomal RNA and cellular RNAs were prepared using an Exosomal RNA Purification kit (Norgen Biotek) and miRNeasy kit (Qiagen) respectively as per manufacturer’s instructions. For FFPE tissues, tumor areas were identified and marked on H & E stained FFPE sections by a board-certified pathologist. Microdissections were performed as described in^58^. RNA were extracted from microdissected FFPE tissues using a miRNeasy FFPE Kit (Qiagen) following the manufacturer’s instructions. The quantity and quality of all RNA preparations were determined by an Agilent Bioanalyzer 2100 (Agilent Technologies) using a nano RNA chip as per the manufacturer’s instructions.

### Small RNA sequencing

Using 50–100 ng of purified RNA, libraries were generated using an Illumina TruSeq small RNA library prep kit (catalog number RS-200-0012) as per manufacturer’s instructions as described in^59^. Set A indices 1–12 and set B indices 13–24 were employed to generate cDNA libraries. Index libraries were equally pooled and sequenced at the institutional molecular core facility using an Illumina NextSeq 500/550 mid output kit (version 2, 150 cycles) as per manufacturer’s instructions. Sequencing reads were adapter trimmed using FASTQC and analyzed by BaseSpace small RNA app (version 1, Illumina). Small RNA app uses MiRDeep* (version 3.2)^60^, DESeq2 (version 1.0.17), SAMtools (version 0.1.19-isis-1.0.2) and Isis analysis software (version 2.5.52.11) for alignment using GCH38 human genome assembly as reference genome. Transcripts were considered significantly differentially expressed if the P-value was < 0.05 and absolute fold change > 2. Heat maps were generated by using R studio software, Version 1.1.463 (https://www.npackd.org/p/rstudio/1.1.463). Unsupervised analysis was performed using Principal Component Analysis (PCA).

### Generation of EV-miRNA-classifier

To develop an optimal EV-based classifier to differentiate between CRPC-NE samples from those with adenocarcinoma histology, differentially expressed miRNAs detected above threshold were used as a seed set. Since n < p (n = number of samples and p = number of variables), to mitigate the effects of this drawback, we employed a combination of techniques. We applied random forest machine learning technique paired with leave-pair-out cross validation (LPOCV) as this methodology has the ability to lessen the effects of high bias/variance^62^. Further, before each cross-validation iteration, Boruta feature selection (https://pypi.org/project/Boruta/) was applied to the training set of a given iteration followed by training the random forest model on the newly subsetted training set. Receiver operating characteristic (ROC) analysis was used for measuring classifier performance wherein area under the curve (AUC) was used as the primary evaluation metric. Detailed description of classifier methods is under supplemental information.

### Mass spectrometric analyses

For mass spectrometry analyses, EVs were isolated from LNCaP-AR, LNCaP-AR-EnzR and NCI-H660 cell lines by total exosome isolation reagent (Life Technologies). Following extensive characterization of EVs, samples were shipped to MS Bioworks (Ann Arbor, Michigan) for mass spectrometric analyses. Protein lysates were prepared by lysing the EVs in 100 μL of 2% modified RIPA buffer combined with protease inhibitors and sonicated. Each sample was incubated at 60 °C for 15 min and clarified by centrifugation. Samples were quantitated by Qubit. 10 μg of each sample was loaded onto a 10% bis–tris SDS-PAGE gel (Novex, Invitrogen) and size-separated. Gel was stained with Coomassie and the entire mobility region was excised into one segment. Samples were digested with trypsin and analyzed on a nano LC/MS/MS with a Waters nanoacquity HPLC system interfaced to a ThermoFisher Q exactive. Differentially expressed proteins were further analyzed using the Database for Annotation, Visualization and Integrated Discovery (DAVID) v 6.8 software^63^ to discover their association with biological processes, cellular components and KEGG pathways^36,37^.

### Immunoblotting analyses

Exosomes were lysed with RIPA buffer [50 mmol/L Tris (pH 8.0), 150 mmol/L NaCl, 0.5% deoxycholate, 0.1% SDS, and 1.0% NP-40] containing protease inhibitor cocktail (Roche). Protein lysates (20 μg) were loaded onto a 4–20% Tris–glycine gradient gel (Biorad), transferred onto PVDF membranes and Western blotting was performed as per standard protocols. Following transfer, membranes were cut according to predicted molecular sizes and probed with following antibodies: THBS1 (Cell Signaling Technology, 37879), CD9 (Cell Signaling Technology, 13174), CD63 (System Biosciences, EXOAB-CD63A-1), Alix (Cell Signaling Technology, 2171), TSG101 (System Biosciences, EXOAB-TSG101-1) and Gelsolin (Cell Signaling Technology, 12953).

### Equipment and settings

All the Western blot images were developed on X-ray films using Konica Minolta Medical film processor (SRX-101) with the default settings. The X-ray film was exposed for 2 min for TSG101, Gelsolin and Thrombospondin bolts and and 1 h for CD63 blots. The developed films were scanned using Bizhub C364e scanner with final scans with following settings: dimensions 1700 × 2200, resolution 96 dpi and bit depth 24. The scanned images were used as is for the final figure without any other image processing changes.

### Quantitative real-time PCR

Mature miRNAs were assayed using the TaqMan microRNA expression assays (Applied Biosystems) in accordance with the manufacturer's instructions. TaqMan assays used were miR-28-3p (TM002446), miR-148a-3p (Tm000470) and U6 (TM001973). The comparative Ct method was used to calculate the relative changes in gene expression on the 7500 Fast Real Time PCR System.

### Thrombospondin 1 ELISA

TSP1 ELISA was performed using an ELISA kit (ThermoFisher Scientific, Cat no. BMS2100) as per manufacturer’s instructions.

### Statistics

All quantified data represents an average of triplicate samples or as indicated. Data are represented as mean ± S.E.M or as indicated. Statistical analyses were performed using MedCalc version 10.3.2 or R package (https://www.r-project.org/). Statistical significance between groups was assessed by two-tailed Student's t-test. Results were considered statistically significant at P ≤ 0.05.

## Supplementary Information

Supplementary Information 1.

Supplementary Information 2.

Supplementary Information 3.

Supplementary Information 4.

Supplementary Information 5.

Supplementary Information 6.
