# Supplementary material for: Novel, non-invasive markers for detecting therapy induced neuroendocrine differentiation in castration-resistant prostate cancer patients
Source: Sci Rep. 2021 Apr 15;11:8279. doi: 10.1038/s41598-021-87441-2 (PMC8050049; doi:10.1038/s41598-021-87441-2)

Novel, non-invasive markers for detecting therapy induced neuroendocrine differentiation in castration-resistant prostate cancer patients

Divya Bhagirath[1], Michael Liston[2], Theresa Akoto[3], Byron Lui[2], Barbara A. Bensing[2], Ashok Sharma[4] and Sharanjot Saini[1]

[1]Department of Biochemistry and Molecular Biology, Augusta University

[2]Veterans Affairs Medical Center, San Francisco

[3]Department of Cellular Biology and Anatomy, Augusta University

[4]Department of Population Health Sciences, Augusta University

SUPPLEMENTARY METHODS

Classifier Methods[1]

Tools used: Python – Scikit-learn, Numpy, Pandas, BorutaPy

Outline:

I. **Data Intro**
   a. Features:
      i. *Phase 0*: Original Data set
      ii. *Phase 1*: Filtered Data set – (filtering was done based on **(1)** p-value column (0.05), **(2)** status column (Low, OK, Outlier), and **(3)** miRNA vs. ISO-miRNA.
      iii. *Phase 2*: ISO-mIR features filtered out.
   b. Observations:
      i. 27 total observations; 21 = Control, 6 = Test
      ii. Labels were generated based on Control, Test categorization (Control = FALSE, Test = TRUE).

II. **Data Cleaning**
   a. Filtering (as detailed in section ii of Data Intro, Dimensions)
   b. Transpose dataframe
      i. The data was initially presented with features as rows and observations as columns.

III. **Feature Selection**
   a. BorutaPy library
      i. Feature selection wrapper built around Random Forest algorithm.
      ii. Forest tree depth for BorutaPy feature selection = 6 (Suggested tree depth from documentation: 3 to 7)

IV. **Classifier Setup/Cross Validation**
   a. Run 1: Random Forest with LPOCV
      i. Purpose:
         1. Evaluate Random Forest model performance in conjunction with LPOCV.

      ii. LPOCV setup:

         1. Compute all possible pair combinations of positive/negative classes (21 negative and 6 positive = 21 x 6 = 126 cross validation folds).
         2. Iterate through combinations one by one:

a. Generate data subset (training/validation sets)
b. Feed training set into BorutaPy feature selection.
c. Based on features suggested by BorutaPy, subset training/validation sets again.
d. Train Random Forest model on new training set.
e. Run predict function on validation pair (output are prediction probabilities from Random Forest predict_proba function).
f. REPEAT until all 126 leave pair out cross validation folds are complete.

b. Evaluation
   i. Pooled AUC Calculation
      1. Using the probability outcomes from all 126 cross validation iterations, area under the curve was calculated using Scikit-Learn's roc_auc_score metric (based on trapezoidal rule).

   ii. Average AUC Calculation
      1. Calculate area under the curve score for each validation pair.
      2. Average all 126 calculations.

   iii. Feature Importance
      1. Ranking of each features' average importance across all 126 models trained.

SUPPLEMENTAL FIGURE LEGENDS

Fig. S1 Principal Component Analyses for CRPC-Adeno and CRPC-NE based on piRNAs

Unsupervised principal component analyses (PCA) based on differential expression of piRNAs, as performed in CRPC-adeno cases (n=21) and CRPC-NE models (n=6 + NCI-H660 cell line).

Fig. S2 Characterization of EVs isolated from prostate cancer cell lines

Representative NTA analyses for LNCaP-AR (left panel), LNCaP-AR EnzR (middle panel) and NCI-H660 (upper right panel) cell lines. Particle concentrations as determined by NTA analyses are listed.

SUPPLEMENTARY TABLE LEGENDS

Table S1 Clinicopathologic characteristics of metastatic CRPC patients

Table summarizing the age, race, Gleason score of primary tumor, final serum PSA, metastatic sites and prior therapies of CRPC-Adeno and CRPC-NE samples (treatment-induced NEPC) used in the study.

Table S2 MicroRNA expression in CRPC Adeno vs CRPC NE EVs

Table showing significantly dysregulated miRNAs identified by sequencing of serum EVs isolated from CRPC-Adeno (n=21) and CRPC-NE clinical samples (n=6) and NCI-H660 cell line.

Table S3 MicroRNAs altered in PCa EVs and corresponding clinical tissues

Table showing corresponding tissue expression of significantly dysregulated miRNAs identified by sequencing of EVs isolated from CRPC-Adeno vs CRPC-NE clinical samples.

Table S4 List of miRNAs altered in *de novo* NEPC

Table showing significantly dysregulated miRNAs identified by sequencing of prostate adenocarcinomas vs *de novo* NEPC cases.

Table S5 List of proteins identified by mass spectrometric analyses of protein content of EVs from NEPC cellular models

Following extensive characterization of EVs, proteins were isolated from LNCaP-AR, LNCaP-AR-EnzR and NCI-H660 cells followed by mass spectrometric analyses by Shot gun approach. List of proteins identified are represented.

Table S6 Bioinformatic analyses of mass spectrometry data

Examination of protein content of EVs from LNCaP-AR, LNCaP-AR-Enz resistant and NCI-H660 cell lines by mass spectrometric analyses identified several differentially expressed proteins in

LNCaP-AR-ENZ resistant and NCI-H660 EVs as compared to LNCaP-AR EVs (Fig. 6 and Table S5). List of KEGG pathways[2,3] (sheet 1), cellular fractions (sheet 2) and molecular functions (sheet 3) of proteins isolated from EVs of NCI-H660 cells as compared to EVs from LNCaP-AR cells as shown by the Database for Annotation, Visualization and Integrated Discovery (DAVID) v 6.8 software [4]. *In silico* analyses of cellular processes impacted by identified altered EV proteins showed that focal adhesion, phagosome, ECM-receptor interactions, complement and coagulation cascades and glycolysis/gluconeogenesis are top processes impacted by altered protein in NCI-H660 EVs (sheet 1 of table S6 and Fig. 6A). Further, 35% of EV proteins found in NEPC exosomes were predicted to be membranous, 56% cytoplasmic, 55% cytosolic, 38% nuclear, 20% were found to associated with cell cell adherens junction, 16% with focal adhesion and 13% were cell surface proteins (sheet 2 of table S6 and Fig. 6B). *In silico* analyses of impacted biological processes showed that proteins involved in cell-cell adhesion, protein stabilization, protein folding, extracellular matrix organization and negative regulation of apoptotic process were highly represented (sheet 3 of Table S6 and Fig. 6C).

SUPPLEMENTARY REFERENCES

1 Bhagirath, D. *et al.* MicroRNA determinants of neuroendocrine differentiation in metastatic castration-resistant prostate cancer. *Oncogene*, doi:10.1038/s41388-020-01493-8 (2020).

2 Kanehisa, M. Toward understanding the origin and evolution of cellular organisms. *Protein Sci* **28**, 1947-1951, doi:10.1002/pro.3715 (2019).

3 Kanehisa, M. & Goto, S. KEGG: kyoto encyclopedia of genes and genomes. *Nucleic Acids Res* **28**, 27-30, doi:10.1093/nar/28.1.27 (2000).

4 Huang da, W., Sherman, B. T. & Lempicki, R. A. Systematic and integrative analysis of large gene lists using DAVID bioinformatics resources. *Nat Protoc* **4**, 44-57, doi:10.1038/nprot.2008.211 (2009).

Fig. S1 Principal Component Analyses for CRPC-Adeno and CRPC-NE based on piRNA profile of corresponding EVs

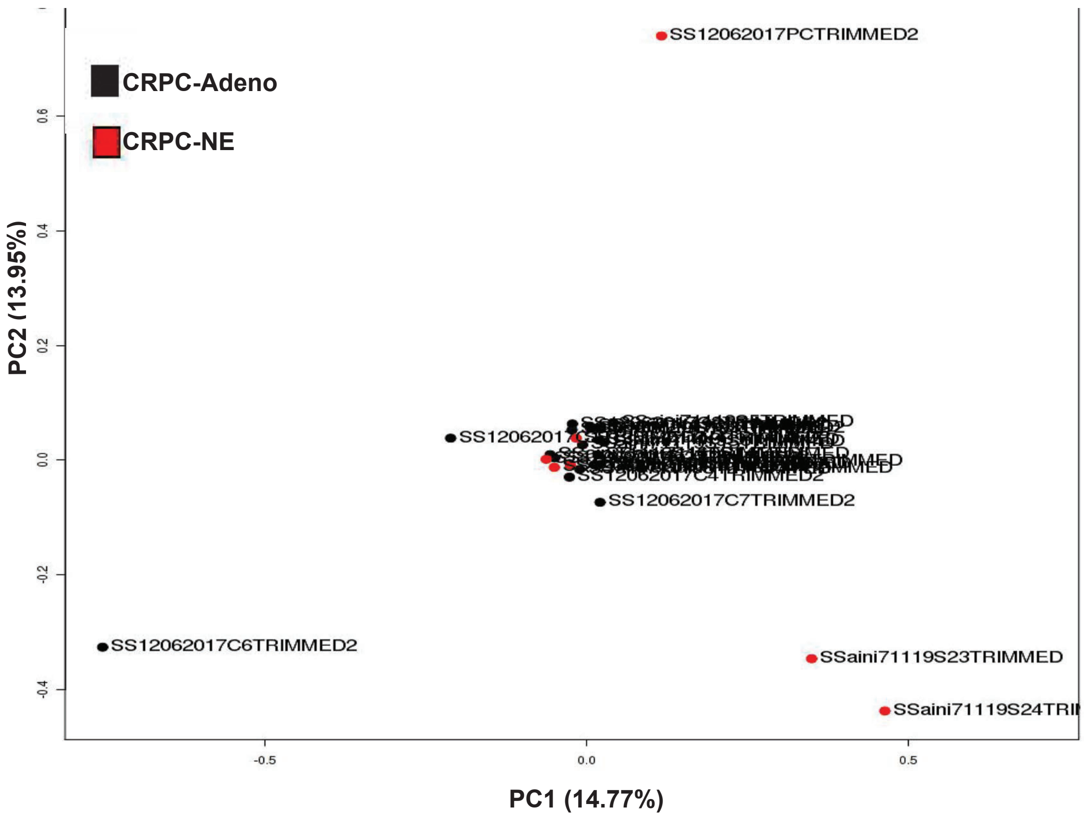

Fig. S2 Charcaterization of exosomes/EVs isolated from prostate cancer cell lines

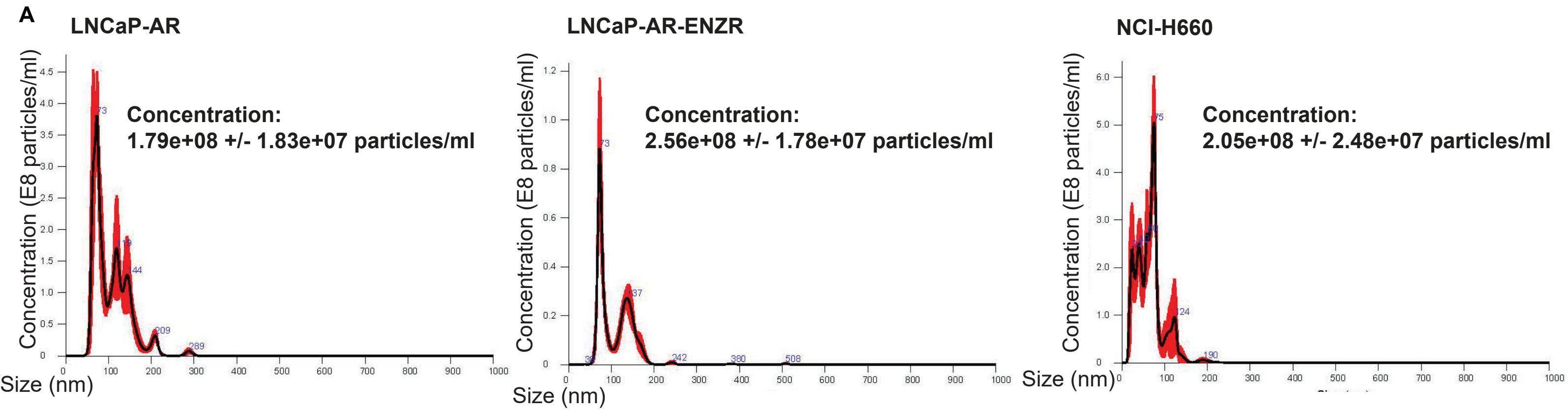

Table S1. Clinicopathologic characteristics of prostate cancer patients

**Patient Characteristics**	**CRPC (Adeno)**	**CRPC (NE)**
Number of patients	22	6
Median age at diagnosis in years (range)	64 (45 - 80)	62 (57 - 64)
Median age at death in years (range)	72 (90+ - 53)	67 (60 - 76)
Median survival after diagnosis in years (range)	7 (2 - 25)	4 (2 - 13)
Median PSA at death in ng/mL (range)	328.85 (5.63 - 15000)	1 (0.15 – 8.85)
Race: Caucasians **(C)** Others including African American, Hispanics, Asians **(O)**	**C**- 20 **O**- 2	**C**- 5
**Gleason Score**		
4-6	3	-
**7**	8	2
8-10	9	3
Unknown	2	-
**Metastatic sites**		
Lung	3	1
Liver	5	1
Lymph node (LN)	7	-
Others (Skin, Retroperitoneal, Periaortic LN, Retroperitoneal LN, Diaphragm, Retrosternal LN, Spleen, Omentum, Cortical, Pelvic LN, Mass)	7	3
**Androgen Ablation Therapy**		
Number of patients receiving androgen ablation therapy (%)	22 (100)	5 (100)

Number of patients receiving Enzalutamide, Abiraterone or both (%)	3 (14)	1 (20)
**Other Therapies**		
Ketoconazole (%)	10 (45)	1 (20)
DES (%)	8 (36)	1 (20)
Corticosteroids (%)	15 (68)	3 (60)
Estramustine (%)	4 (18)	1 (20)
Taxotere (%)	14 (64)	3 (60)

Western blots Fig. 1

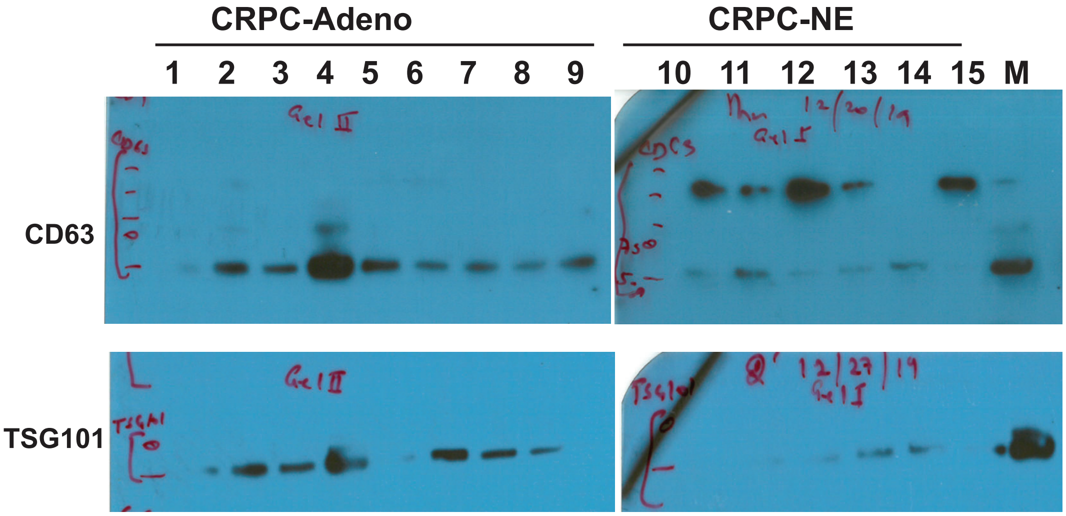

Western blots Fig. 5

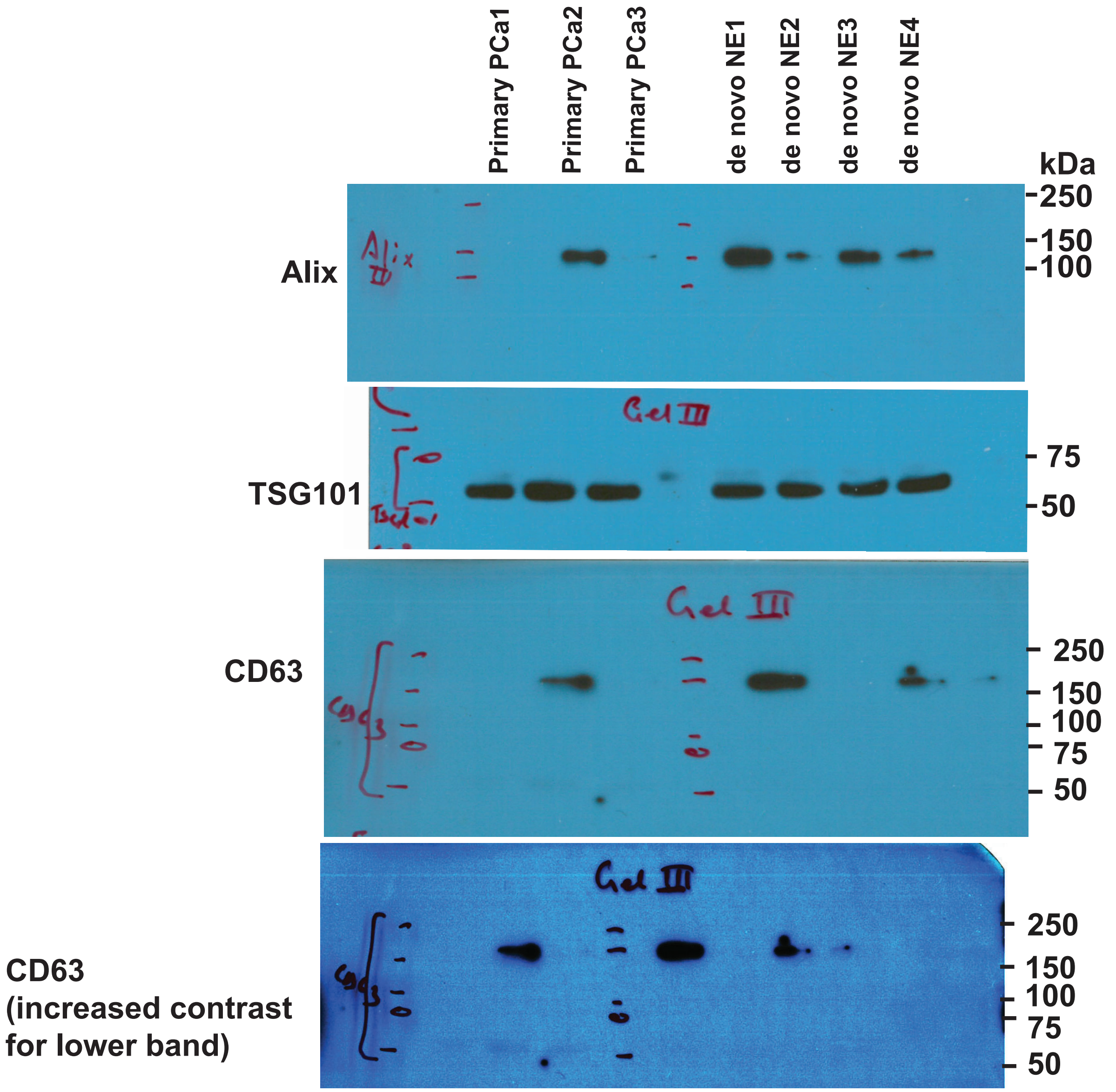

Western blots Figure 7

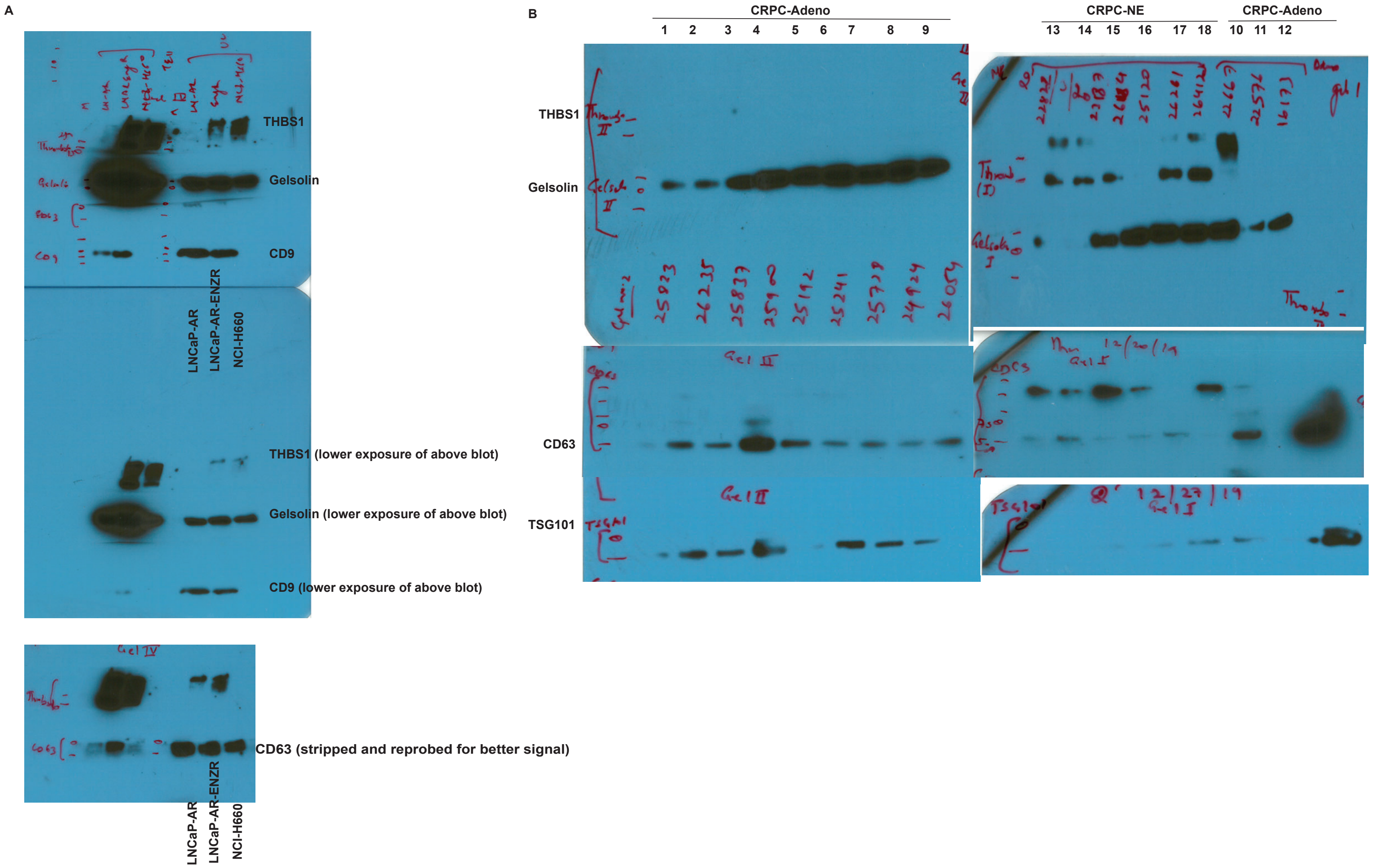

Supplement: Supplementary file 1 — Supplementary Information 1. [file 41598_2021_87441_MOESM1_ESM.pdf]
